# Peripheral Primitive Neuroectodermal Tumor of the Pelvis

**Published:** 2014-01

**Authors:** Zohreh Yousefi, Nourieh Sharifhi, Malihe Hasanzadeh, Mansoureh Mottaghi, Somayeh Bolandy

**Affiliations:** 1 Cancer Research Center, Ghaem Hospital, School of Medicine, Mashhad University of Medical Sciences, Mashhad, Iran;; 2 Department of Pathology, Ghaem Hospital, School of Medicine, Mashhad University of Medical Sciences, Mashhad, Iran;; 3 Department of Obstetrics and Gynecology, Ghaem Hospital, School of Medicine, Mashhad University of Medical Sciences, Mashhad, Iran;; 4 Gynecologist, Mashhad, Iran

**Keywords:** Primitive neuroectodermal tumors, Ewing’s sarcoma, Pelvic neoplasm, Ovary, Broad ligament

## Abstract

The primitive neuroectodermal tumor (PNET) belongs to a group of highly malignant tumors and is composed of small round cells of a neuroectodermal origin. Categorized in the same tumor family as Ewing sarcoma, the PNET is most likely to occur in bones and soft tissues. However, a small number of PNET cases arising in the pelvis have been reported as well.

We present three cases of pelvic PNET: two cases in the ovary and one case in the broad ligament. The PNET often exhibits aggressive clinical behavior with worse outcomes than other small round cell tumors. The significant prognostic factors of the PNET include site of tumor, volume of neoplasm, and presence of metastasis. The treatment protocol is multimodal and includes local surgical treatment followed by chemotherapy.

We herein describe three PNET cases as a rare entity in the pelvis. Pelvic PNETs should be included in the differential diagnosis of pelvic masses.

## Introduction

The primitive neuroectodermal tumor (PNET) of the female genital tract is a rare entity. The term “PNET” was first used by Hart and Earle in 1973 to introduce a group of tumors derived from fetal neuroectodermal cells.^1^ According to the cell of origin and location, two main classifications of the PNET include central and peripheral. A group which often involves the sympathetic nervous system or soft tissues and bones is described as the peripheral PNET; this group arises from the neural crest and primitive neuroendocrine cells.^2^ Risk factors for the PNET have yet to be clearly identified.^3^ Pelvic PNETs are usually observed in the uterine corpus, ovaries, cervix, and vulva.^4^^,^^5^ Also, the literature contains rare cases of the PNET in the broad ligament.^6^

Conventionally, the diagnosis of the PNET can be made based on histological examinations. However, immunohistochemical profiles and genetic studies commonly help the pathologist to differentiate between the PNET and other small round cell tumors. Immunohistochemically, the PNET is frequently reactive for vimentin, HMWCK, and CD-99.^7^ Radiological studies such as Computed Tomography (CT) scan and Magnetic Resonance Imaging (MRI) are essential in the diagnosis of tumor involvement and ruling out of metastatic disease. A review of CT and MRI findings in PNET cases suggests that no characteristic finding aids in the preoperative diagnosis of this tumor. Clinical manifestations depend on the surrounding structures on account of mass effect. Treatment option for the PNET includes surgical resection of the tumor, followed by additional chemotherapy. After successful chemo or/and radiotherapy, the 5-year survival rate is only 7.6-8%.^8^ A review of literature reported that the 2-year disease-free survival rate in patients with only localized disease is 25%.^9^ Fortunately, advances in diagnostic modalities and neoadjuvant and adjuvant chemotherapeutic regimens may be improving long-term disease-free survival.

We herein present three unusual cases of the PNET arising in the pelvis.

## Case Reports

**Figure 1:**
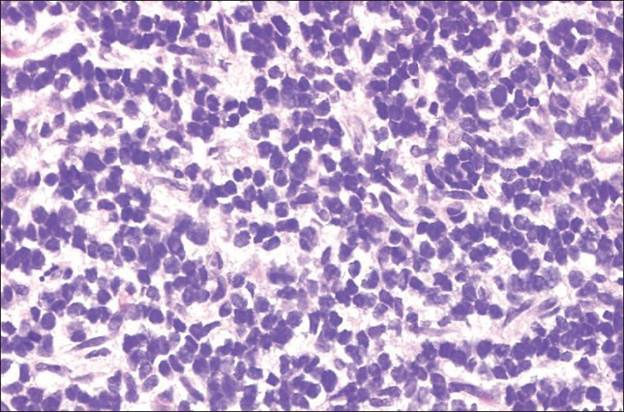
The primitive neuroectodermal tumor with strong immunoreactivity for CD99, vimentin, and CK5 markers.

The first patient was a 21-year-old woman (G_2_A_1_) who referred to the Tumor Clinic of Ghaem Hospital. The patient’s history included two month’s pregnancy with abortion.Pelvic ultrasonography revealed , mixed, echogenic mass in the left adnex (mean diameter=102×68 cm) with multiple internal septation and adhesion to the surrounding organs. The concentrations of all tumor markers were normal (alpha-photo protein=8, B HCG=1, LDH=410, and CA-125=30). Pelvic examination detected a palpable, firm, irregular mass in the posterior vaginal fornix. An exploratory laparotomy was performed, and a predominant mass (about 20×15 cm in diameter) situated in the pelvis in the broad ligament was observed. The tumor was soft and friable with hemorrhagic, necrotic, and cystic lesions. The uterine serosa was coated with the tumor. The tumor infiltrated most of the posterior peritoneal wall. After the resection of the tumor, frozen-section analysis identified a malignant tumor, probably lymphoma of the pelvis. The medical oncology consultant required bone marrow aspiration, which showed normocellular bone marrow without evidence of metastatic involvement. Additional metastatic work-up yielded a negative bone scan. Permanent sections and immunohistochemical profile with positive immunoreactivity for HMWCK, vimentin and CD-99 as well as negative immunostaining for LCA, desmin, NSE, and chromogranin allowed the pathologist to distinguish the PNET from other small round cell tumors (figure 1). Because it was impossible to perform complete surgery, a chemotherapy regimen was fashioned using aggressive multi-agent chemotherapy, including the BEP regimen (Bleomycin, Cisplatinum, and Etoposide). The patient, however, died due to pulmonary metastasis 3-4 months after diagnosis.

**Figure 2:**
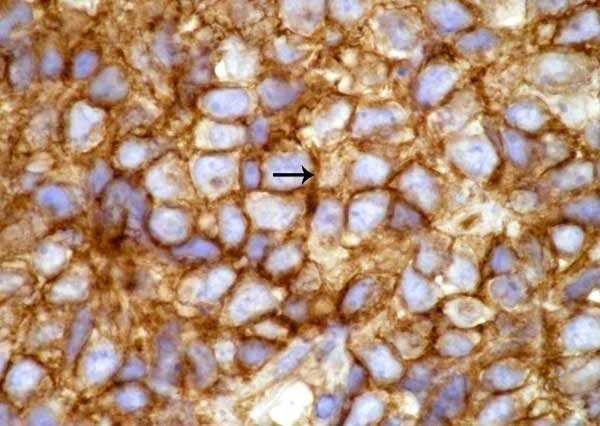
The primitive neuroectodermal tumor with strong immunoreactivity for CD99.

The second patient was a 24-year-old virgin with 10kg weight loss and dull aching in the lower abdomen. She had suffered from weakness in the lower extremity for the past three months. Physical examination revealed an irregular, non-tender mass (30×35 cm in diameter), involving the left lower abdomen with extension to the xiphoid. Pelvic ultrasonography revealed a multi-loculated, echogenic mass in the pelvis and abdomen, and CT-scan identified a large retroperitoneal mass with liver metastasis (figure 2). The CA-125 concentration was elevated at 412, and the level of LDH was 5911, but the other tumor markers were normal. The initial appearance of the tumor suggested a diagnosis of ovarian carcinoma. In exploratory laparotomy, an ovarian mass with friable attachment to pelvic peritoneal surfaces was encountered. Frozen section identified the malignant tumor as probably a granulose cell tumor. Surgical staging of ovarian cancer with fertility-sparing surgery was performed. During laparotomy, a great deal of unusual lymphadenopathy was observed in the pelvic and paraaortic area. Further permanent pathology analysis of the surgical specimen and immunohistochemistry showed negative immunoreactivity for inhibin and revealed an undifferentiated malignant neoplasm, consisting of malignant small round cells with monomorphic nuclei and scant cytoplasm with features most consistent with the PNET. Immunohistochemical profile, positive immunoreactivity for CD99, CK, and vimentin were suggestive of the PNET. Ten days after surgery, because of the patient’s headache and paresthesia in the extremities, MRI was performed and detected a heterogeneous mass in T4 and T5. She underwent emergency laminectomy due to head and neck metastasis. The patient was subsequently treated with chemotherapy (BEP regimen), but she died due to brain metastasis before completing all the courses of chemotherapy.

**Figure 3:**
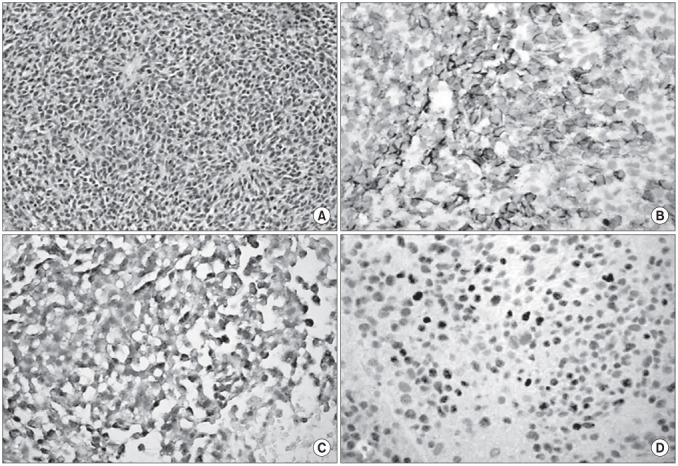
a): The primitive neuroectodermal tumor (PNET) with negative immunoreactivity for LCA. b): PNET with negative immunoreactivity for NSE. c): PNET with negative immunoreactivity for chromogranin. d): PNET with negative immunoreactivity for desmin.

The third patient was a 43-year-old woman (G3 P3) with a history of pelvic pain and distention in the hypogastric area. In pelvic examination, a large, solid, round mass (about 25×30 cm in diameter) with extension to the umbilical area was detected. Pelvic ultrasonography revealed a well-defined, mixed, echogenic mass (mean diameter=22×35 cm) in the right adnex with internal echo with adhesion to the surrounding organs and little ascetics. All tumor markers were normal (CA-125=25 and CEA=4). Exploratory laparotomy showed an irregular, solid mass (about 20×30 cm in diameter) in the right ovary that had infiltrated the other organs of the pelvis. Frozen section reported a malignant tumor, most probably adenocarcinoma of the ovary. Accordingly, surgical staging surgery was performed. Permanent sections and immunohistochemical profile were compatible with the PNET (figure 3). Four weeks after surgery and before commencement of chemotherapy, the patient was admitted to the Emergency Ward with tachycardia and dyspnea. She died from pulmonary metastasis several days after diagnosis.

## Discussion

The PNET accounts for 1% of all soft tissue sarcomas and occurs even less frequently in the female reproductive tract. Nearly 80% of the patients are younger than 20 years.^10^ Most cases of the PNET occur in the soft tissue; however, rare cases have been reported in the ovary. Therefore, the PNET must be considered in the differential diagnosis of an unusual tumor in the pelvis, particularly in young patients.^11^ Clinical symptoms, including pain and swelling of the surrounding organs, are related to the location of the tumor. All of our patients had no specific sign and symptom recommended for the diagnosis of a pelvic PNET.

In 99% of PNET cases, the reciprocal translocation t(11;22) is observed in cytogenetic examination. We, however, were not able to ascertain this in our patients because our center lacks the required means for this technique. 

In general, the PNET is a very highly aggressive tumor with tendency to extension out of the pelvic cavity. Patients may have metastatic disease at the first visit, so a full metastatic work-up is indicated in a suspected case of the PNET.^12^ The most common sites of PNET metastases are the lung, bone, and bone marrow.^13^ One of our patients had lung metastasis and in the others brain metastasis was encountered in the early postoperative period.

Surgical management of the PNET includes complete resection of the tumor with an effort for debulking if possible. By reducing the tumor size, we can reduce the potential reservoir of drug-resistant tumors. Nevertheless, complete surgical resection of these tumors is not possible due to the aggressive behavior and dissemination of the neoplasm.^14^ In one of our patients, we did not succeed in performing complete surgical resection. Also, in the other two patients, we found complete pelvic and paraaortic lymphadenectomy impossible.

Chemotherapy regimens have significantly improved outcomes in patients with the PNET. A postoperative chemotherapy program as an adjuvant therapy is recommended immediately following diagnosis. Many centers utilize the same chemotherapy regimens that are used for gem cell tumors. In the current treatment protocols, chemotherapy regimens encompass a combination of Vincristine, Actinomycin D, Cyclophosphamide, and Doxorubicin. Moreover, a combination of Ifosfamide and Etoposide appears to offer the greatest survival advantage. The prognostic factors of the PNET are dependent on the site, volume, and extension of the tumor.^15^All of the three PNET patients in this report had very poor prognoses and died before complete postoperative chemotherapy.

## Conclusion

The PNET is an aggressive, malignant tumor and should be considered in the differential diagnosis of pelvic masses.
